# *Echinocephalus caniculus* n. sp. (Nematoda: Gnathostomatidae Railliet, 1895) from the lesser spotted dogfish *Scyliorhinus canicula* (L.) (Elasmobranchii: Scyliorhinidae Gill, 1862) off Tunisia, with a key to species of the genus *Echinocephalus*

**DOI:** 10.1007/s11230-022-10027-7

**Published:** 2022-03-14

**Authors:** Chiraz Ben Saad, Jaydipbhai Suthar, Stefan Theisen, Harry W. Palm, Lamia Gargouri

**Affiliations:** 1 Laboratory of Diversity, Management and Conservation of Biological Systems, Faculty of Sciences of Tunis, University of Tunis El Manar, LR18ES06, Tunis, Tunisia; 2 Aquaculture and Sea-Ranching, Faculty of Agricultural and Environmental Sciences, University of Rostock, Rostock, Germany

## Abstract

*Echinocephalus caniculus* **n. sp.** (Nematoda, Gnathostomatidae Railliet, 1895) was isolated from the spiral valve of the lesser spotted dogfish *Scyliorhinus canicula* (L.) from the waters off Kalaat El Andalous, North East Tunisia. This new species is mainly characterized by a cephalic bulb armed with 31–39 transverse rows of uncinated hooks, a comparatively long oesophagus, short spicules and the presence of a gubernaculum. The new species differs from its congeners by having four cervical sacs of almost equal length, a higher oesophagus/body length ratio, the arrangement of the caudal papillae, the absence of a medioventral preanal organ and numerous scattered `pores´ limited to the lateral side of the posterior part of the body. This is the first report of a member of the genus *Echinocephalus* Molin, 1858 from the Tunisian coast, and a new host and locality record for the Gnathostomatidae. A key to the species of *Echinocephalus* is provided.

## Introduction

*Echinocephalus* Molin, 1858 is a nematode genus whose members are found in the gastro-intestinal system of sharks and rays worldwide (Baylis & Lane, 1920; Soota, 1983; Moravec & Justine, 2006). The biology and life cycle of the species of *Echinocephalus* are poorly understood, although larval stages of this genus are known to infect sea urchins (Pearse & Timm, 1971), oyster (Cheng, 1975) and bivalves (Moazzam & Moazzam, 2014). Obligate intermediate hosts of *Echinocephalus* spp. are probably marine crustaceans (copepods), whereas sea urchins, bivalves and teleosts serve as paratenic hosts only (or possibly as the second intermediate hosts) (Ivashkin & Khromova, 1976; Anderson, 2000; Moravec & Justine, 2021). It has been postulated that *Echinocephalus* species can be considered zoonotic (Hoberg et al., 1998), possibly involving a health risk for humans (Miyazaki, 1960; Brooks & Deardorff, 1988; Jeremiah et al., 2011; Shamsi & Sheorey, 2018; Shamsi et al., 2021) when accidentally infected whilst consuming molluscs (Millemann, 1951, 1963). Ko et al. (1975) demonstrated that third stage larvae of *E. crassostreai* Cheng, 1975 [*species inquirenda* (Moravec & Justine, 2021)] from oysters, fed to a cat and a monkey penetrated the stomach and intestines of both animals.

The objective of this study is to describe a new species of *Echinocephalus* of the lesser spotted dogfish *Scyliorhinus canicula* off Kalaat El Andalous, NE Tunisia.

## Materials & methods

Between 6^th^–23^rd^ April 2019, 17 specimens of *Scyliorhinus canicula* were collected by fishermen from the Sea of Kalaat El Andalous, Gulf of Tunis (37°07'23.8"N 10°23'40.7"E), NE Tunisia. Fish were transported on ice directly to the Laboratory of Diversity, Management and Conservation of Biological Systems, Faculty of Sciences of Tunis, University of Tunis El Manar, Tunisia and studied for endohelminths. Fish nomenclature and classification followed Froese and Pauly (2020).

Six nematodes were found alive inside the spiral valve of the infected host, washed in physiological solution and stored in vials containing 70% ethanol. Morphometric studies were performed on 4 specimens (2 males and 2 females) while another 2 were used for Scanning Electron Microscopy (SEM). For light microscopic examination, 2 males and 2 females were cleared in lactophenol. Drawings were made with a drawing tube attached to an Olympus BX53 DIC microscope. For SEM, samples were dehydrated in an ascending series of ethanol and transferred to 100% acetone, critical point dried, mounted on aluminium SEM-carrier with adhesive conductive carbon tape (PLANO) and coated with gold (10 nm–20 nm layer) under vacuum (EM SCD 004, BALTE). Nematodes were analysed by a field emission scanning electron microscope (FE-SEM, MERLIN^®^ VP Compact, Zeiss), and SEM-images were taken from the selected regions (conditions like applied detector, accelerating voltage, magnification, working distance are given in the figures). All measurements are in micrometers unless otherwise stated.

## Results

**Gnathostomatidae Railliet, 1895**

**Gnathostomatinae Railliet, 1895**

***Echinocephalus*** Molin, 1858 (type species**:** *Echinocephalus uncinatus* Molin, 1858; original designation)

**Fig. 1:**
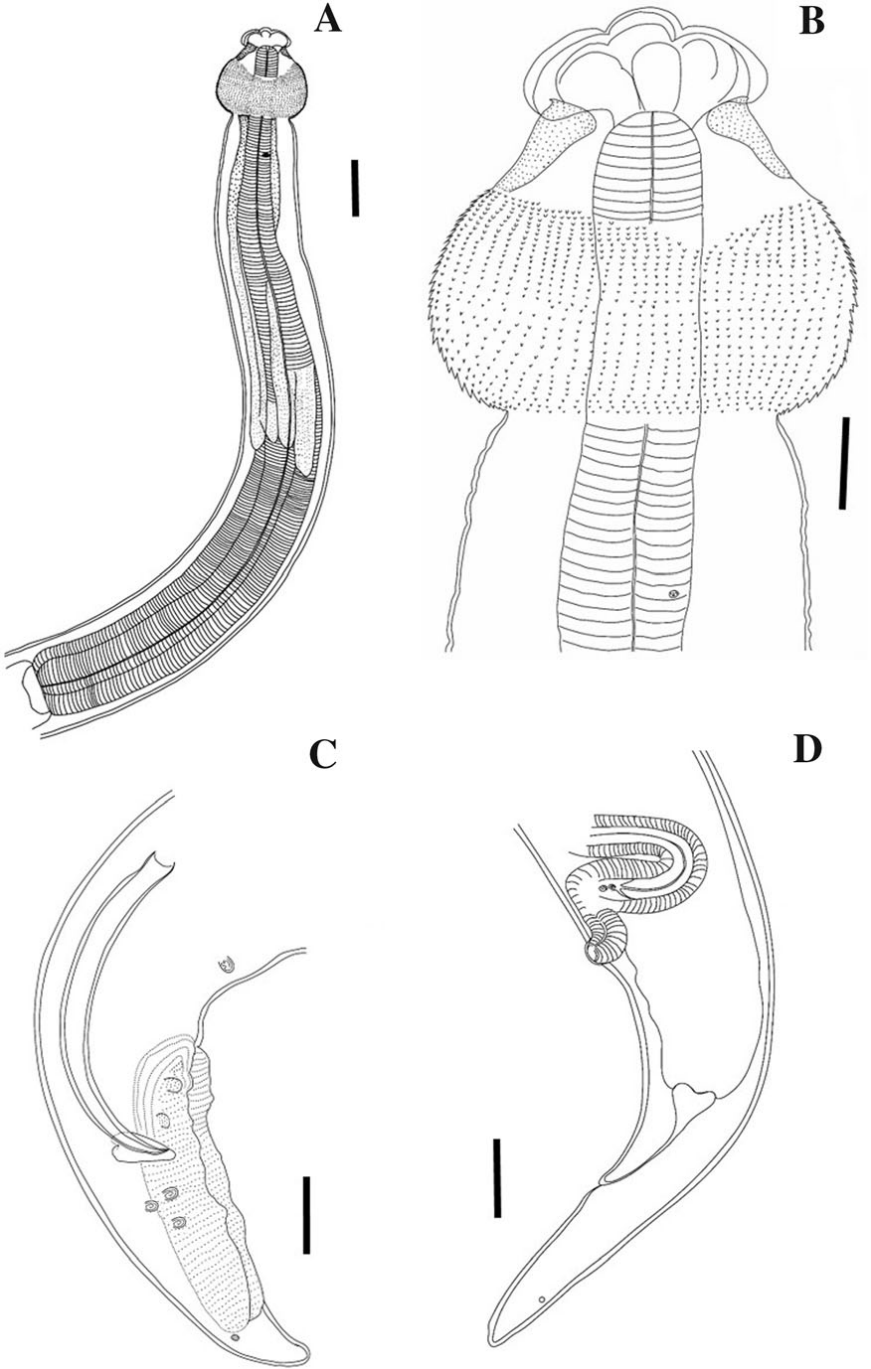
*Echinocephalus caniculus* **n. sp.**(A): Anterior part of female, lateral view (scale bar: 500 µm); (B): Anterior extremity, lateral view (scale bar: 200 µm); (C): Caudal end of male, lateral view (scale bar: 200 µm); (D): Posterior end female with vulva, lateral view (scale bar: 300 µm)

**Fig. 2:**
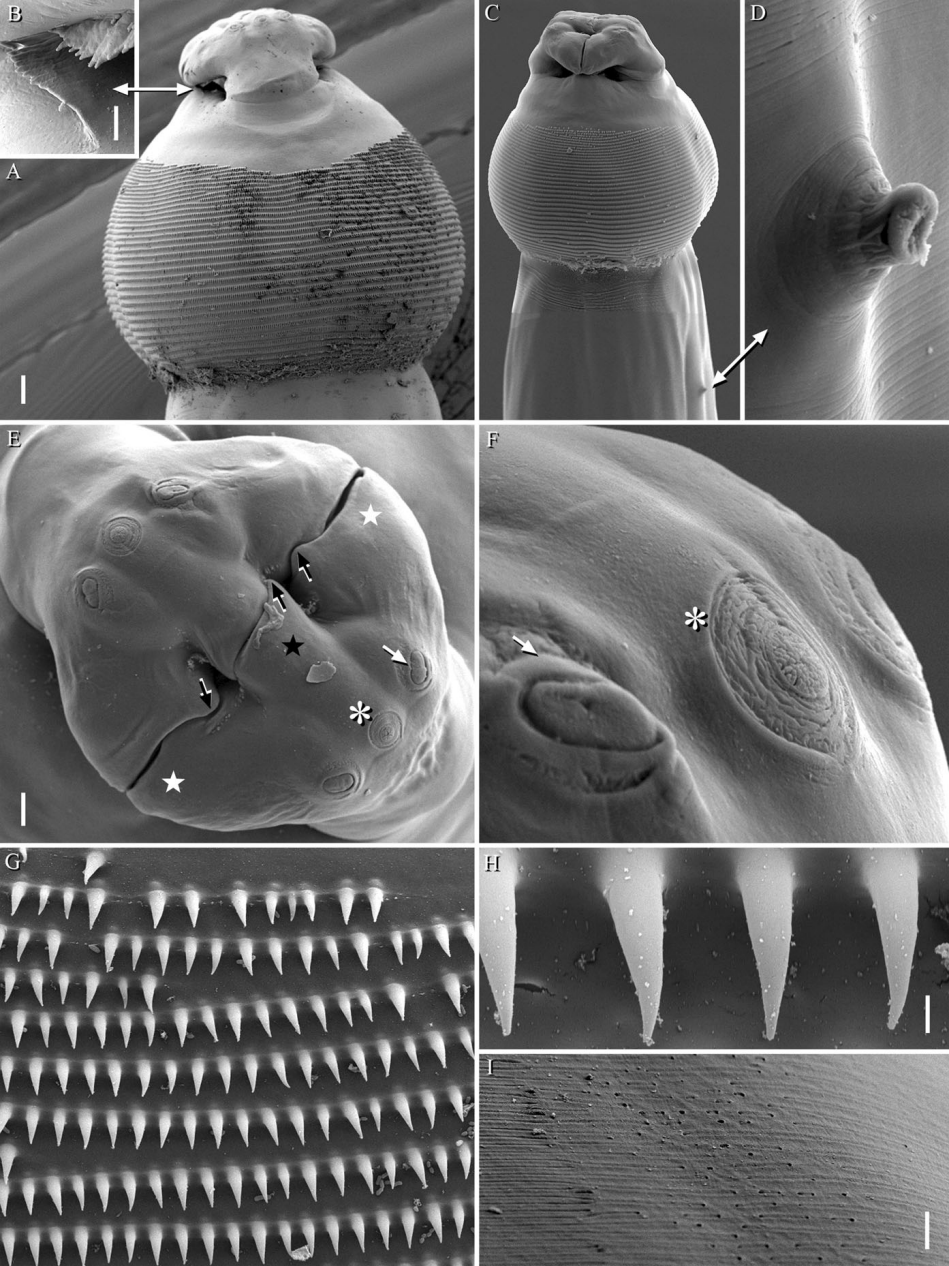
*Echinocephalus caniculus* **n. sp.** SEM micrographs of male (A): Anterior end, lateral view (scale bar: 50 µm); (B): Detail of interlabia (scale bar 5: µm); (C): Cephalic end, dorsoventral view (scale bar: 100 µm); (D): Deirid (scale bar: 5 µm); (E): Apical view of cephalic end/pseudolabia (asterisk: amphid, white arrow: cephalic papillae, black arrows: cuticular thickenings, stars: median lobe (black) and lateral lobes (white)) (scale bar: 20 µm); (F): Detail of amphid (asterisk) and cephalic papillae (arrow) (scale bar: 5 µm); (G): Rows of spines on cephalic bulb (scale bar: 5 µm); (H): Hooks (scale bar: 1 µm); (I): Posterior part of male, lateral view, numerous pores-like stricture (scale bar: 5 µm)

**Fig. 3:**
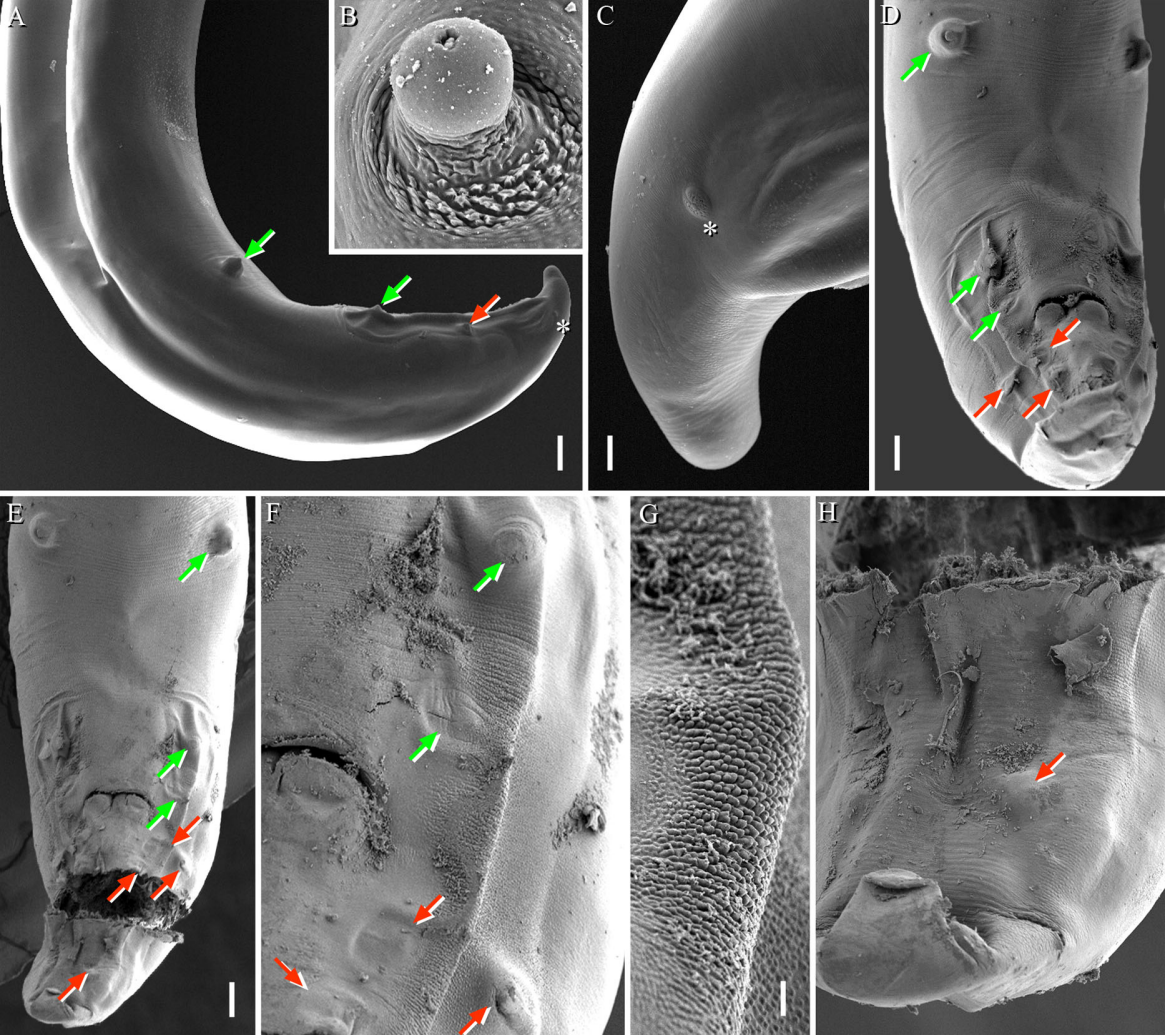
*Echinocephalus caniculu* **n. sp.** SEM micrographs of male (A): Posterior end, lateral view (scale bar: 100 µm); (B): Caudal papilla (scale bar: 5 µm); (C): Tail end, lateral view (scale bar 20: µm); (D&E): Posterior end, ventral view (scale bar: 100 µm); (F): Detail of papillae (scale bar: 20µm); (G): Small cuticular bosses on lateral side of cloaca (Area rugosa) (scale bar: 5 µm); (H): Caudal end, ventral view (scale bar: 20 µm). ***Symbols:*** Asterisk- phasmid, green arrow- precloacal and red arrows-postcloacal papillae

*Echinocephalus caniculus* **n. sp.** (Figs. 1, 2, 3)

*Type-host:* Lesser spotted dogfish *Scyliorhinus canicula* (L.) (Carcharhiniformes: Scyliorhinidae).

*Type-locality:* Kalaat El Andalous, off the Mediterranean coast of North East Tunisia (37°07'23.8"N 10°23'40.7"E).

*Site of infection:* spiral valve.

*Prevalence and intensity:* 6% (1 infected/17 examined); 6 nematodes.

Type-material: Holotype (E.7649), allotype (E.7650) and paratypes [1 male (E.7651) and 1 female (E.7652)] from the same host and locality.

*Depositories:* holotype, allotype and 2 paratypes (male and female)—Berlin Natural History Museum, Germany.

*Etymology:* This species is named after its type-host species name, *canicula* where it was discovered and described for the first time.

**Deposition of type specimens and Life Science Identifiers:**

**ZooBank registration:**

In order to comply with the regulations, set out in article 8.5 of the amended 2012 version of the International Code of Zoological Nomenclature (ICZN, 2012), details of the new species have been submitted to ZooBank. The Life Science Identifier (LSID) for *Echinocephalus caniculus* **n. sp.** is urn:lsid:zoobank.org:pub:AFE7918E-1C87-4592-B9D4-492DD7B7A5B6.

### Description

Body large, dark brown, unarmed except at cephalic bulb. Cuticle with fine transverse striation. Anterior extremity provided with 2 large, equal and massive lateral pseudolabia (Figs. 1A,B, 2A,C,E); each pseudolabium bearing 2 large sublateral/submedian double papillae and a lateral amphid (Figs. 2E,F). Pseudolabia trilobed (1 median, 1 dorsal and 1 ventral lobes); each lobe with cuticular thickenings along external surface (Fig. 2E). Each pseudolabium interlocking with opposite pseudolabium (Fig. 2E). A distinct cuticular striation observed on the dorsal and ventral parts of pseudolabia (Fig. 2B). Interlabia (1 dorsal and 1 ventral) small and serrated (Fig. 2B). Cephalic bulb prominent, armed with 31–39 transverse rows of small, elongated and evenly spaced, posteriorly directed uncinated spines; first few anterior rows of spines incomplete, situated close to each other and non-overlapping (Figs. 2A,G,H). Oesophagus long, not clearly divided into anterior muscular and posterior glandular portions (Fig. 1A). Cervical sacs four, almost equal in length, extending posteriorly and reaching about mid-length of oesophagus (Fig. 1A). Nerve ring and excretory pore not observed. Deirids prominent, well developed and located near posterior part of cephalic bulb (Figs. 1A, 2 C,D). At posterior part of body, numerous lateral pores or pit-like structures scattered in limited area (Fig. 2I). Phasmids paired, located laterally near end of tail (Figs. 1C,D, 3A,C). Tail conical with rounded tip; tip without mucron or any cuticular ornamentation in both sexes (Figs. 1C,D).

**Male (paratype, measurements of holotype in parentheses)**

Body length 26.74 (26.56) mm, maximum width 784 (816). Length of cephalic bulb 436 (531), width at middle part 684 (673). Oesophagus 5.90 (5.97) mm long (22–22% of body length). Cervical sacs 3.19–3.40 (3.19–3.42) mm long. Deirids 879 (1128) from anterior extremity. Spicules equal, 809 (897) long (3–3% of body length) and non-alate. Gubernaculum well-sclerotised, 163 long. Small caudal alae ventrolateral (Figs. 1C, 3A). On either side of cloaca, numerous small cuticular bosses (ornamentation) present (area rugosa not prominent) (Figs. 3F,G), ventral side smooth. Seven pairs of caudal papillae: three sub-ventral precloacal pairs and four pairs of postcloacal papillae of which three pairs subventral and one pair lateral (Figs. 3B,D,E). Caudal papillae not equidistantly distributed. First precloacal pair prominent, situated further away from second pair. A prominent second precloacal pair while third precloacal pair not-prominent situated near each other (Fig. 3F). Three postcloacal pairs close to each other just posterior to cloaca while last pair just in subventral position (Figs. 3F,H). Phasmids 181 from posterior end. Tail 577 long.

**Gravid female (paratype, measurements of allotype in parentheses)**

Body length 40.86 (30.18) mm, maximum width 849 (920). Cephalic bulb 465 (484) long, 751 (749) wide. Oesophagus 6.64 (6.50) mm long (16–21% of body length). Cervical sacs 2.84–3.14 mm long. Deirids 1.09 mm from anterior end. Vulva slightly elevated, located 1.8–2.4 mm from posterior end and at a short distance anterior of anus (Fig. 1D). Vagina anteriorly directed, open into uterus, divided into 2 branches. Uterus didelphic, both branches running parallel anteriorly (prodelphic). Eggs round to oval, thin-walled, 41 long and 33 wide (n= 5). Phasmids located laterally at 304 from posterior end.

### Remarks

The present nematode species belongs to the genus *Echinocephalus* based on the unarmed body, two trilobed pseudolabia, a cephalic bulb provided with transverse rows of spines, a vulva close to the posterior end of the body and a didelphic, prodelphic uterus. So far, the taxonomy of this genus is incompletely resolved due to several incomplete descriptions. For example, six adult species of this genus namely; *Echinocephalus chengii* Singh, Chauhan & Khare, 2010, *E. mastacembeli* Begum & Gupta, 2012 (“it was apparently established on misidentified specimens of *Spinitectus* Fourment, 1884, probably *S. mastacembeli* Karve et Naik, 1951”), *E. mobulae* Kalyankar, 1971, *E. scoliodonti* Lakhsmi, 1994, *E. unispiculus* Arya, 1982 and *E. waltairensis* Lakshmi, Rao & Shyamasundari, 1984 have been reported in India (Moravec & Justine, 2021). However, because of their poor descriptions, they were designated as *species inquirendae* by Moravec and Justine (2021). Moreover, *E. carpiae* Abdel-Ghaffar, Bashtar, Mehlhorn, Abdel-Gaber, Al Quraishy and Saleh, 2013 and *E. crassostreai* were described based on their larval stages only (Cheng, 1975; Abdel-Ghaffar et al., 2013). Larval *Echinocephalus* have undeveloped morphological characteristics compared to adults, e.g., the number of cephalic hooks differs and the reproductive systems are not developed. Moravec and Justine (2021) did not consider *E. carpiae* and *E. crassostreai* as valid species but pointed out their stati as *species inquirendae* or *species dubiae*.

In terms of number of valid species, Bezerra et al. (2020) and Moravec and Justine (2021) recognised 10 and 12 *Echinocephalus* species respectively. Bezerra et al. (2020) did not consider *E. daileyi* Deardorff, Brooks & Thorson, 1981 and *E. diazi* Troncy 1969 as valid species while Moravec and Justine (2021) recognised both these species as valid.

As it possesses more than 30 transverse rows of hooks on the cephalic bulb, the present species differs from *E. multidentatus* Baylis & Lane, 1920, *E. pseudouncinatus* Millemann, 1951 and *E. southwelli* Baylis & Lane, 1920 which have 10–21 rows; and *E. diazi*, *E*. *pteroplateae* Wang et al., 1978 and *E. sinensis* Ko, 1975 which have 25–30 rows. *Echinocephalus pteroplateae* and *E. sinensis* have been reported from the same host order (Myliobatiformes) and zoogeographical region (China) and show similar numbers of transverse rows and length of body (Ko, 1975; Wang et al., 1978). There are minor variations in the distance between the nerve ring, oesophagi and deirids from the cephalic end (considered to be interspecific variation) while other data is not available for further comparison based on their poor descriptions. Therefore, we suggest that *E. sinensis* is a junior synonym of *E. pteroplatea*e and, consequently, we have not included *E. sinensis* in our key to the species. However, Bezerra et al. (2020) and Moravec and Justine (2021) still considered *E. pteroplateae* and *E. sinensis* as separate species.

In possessing more than 30 transverse rows of hooks, the present species is similar to *E. daileyi, E. janzeni* Hoberg, Brooks & Urena, 1998, *E. inserratus* Moravec & Justine 2021, *E. overstreeti* Deardorff and Ko 1987, *E*. *spinosissimus* von Linstow, 1905 and *E. uncinatus* but differs in the combination of different morphological features, host and geographical location (see the key to species). The new species is different from *E. daileyi* (from freshwater stingrays, South America, Deardorff et al., 1981) and *E. janzeni* (from Pacific chupare, *Styracura pacifica* (Beebe & Tee-Van) Hoberg et al., 1998) as it has lower number of caudal papillae (7 pairs vs. 9 pairs), different hosts (Carcharhiniformes vs. Myliobatiformes) and localities (North Africa (Tunisia) vs. Central and South America).

By having interlabia with cuticular striations, the new species can be easily distinguished from *E. inserratus*. Moreover, the new species has been discovered in a different geographical location (North Africa vs. New Caledonia). The new species differs from *E. spinosissimus* by having a lower number of caudal papillae (7 pairs vs. 8 pairs) and a longer distance between the vulva and the tip of the tail (1.8-2.4 mm vs. 1.1-1.4 mm). Similarly, the new species is reported from a different location to *E. spinosissimus* (North Africa vs. Indian Ocean) (Baylis & Dubney, 1920; Beveridge, 1985). By having equal spicules and lower numbers of cloacal papillae, the new species can be differentiated from *E. uncinatus* which has unequal spicules and a higher number of cloacal papillae. Again, the new species has been found in different region (North Africa vs. North Atlantic region) (Beveridge, 1985).

*Echinocephalus overstreeti* most closely resembles the new species but differs in that it has cervical sacs of almost equal length of (vs. unequal), a higher oesophagus-body length ratio (16–22% vs. 11–16%), a ventral surface of the male tail (annulations vs. smooth) and reported different hosts and localities (see the key to species) (Deardorff and Ko, 1983; Beveridge, 1987; Moravec & Justine 2006). Besides, *E caniculus* **n. sp.** differs in possessing numerous pore-like structures scattered laterally on the posterior part of the body.

The new species is also compared with those species that have been reported from similar geographical regions. *E*. *spinosissimus* has been recorded only from the Adriatic Sea (Baylis & Lane, 1920), which is in a similar geographical region to our new species. As mentioned above, *E. caniculus* **n. sp.** is distinct from *E. spinosissimus*.

*Echinocephalus carpiae* [*species inquirenda*], *E. janzeni*, *E. inserraus*, *E. overstreeti* and *E. sinensis* [junior syn. of *E. pteroplateae*] have been studied by scanning electron microscopy (Hoberg et al., 1998; Moravec & Justine, 2006, 2021; Abdel-Ghaffar et al., 2013), illustrating these specimens more precisely. We conclude that there are 12 valid species in the genus *Echinocephalus* and the key to each species is provided below.

**Key to species of the genus** *Echinocephalus* Molin, 1858

1. Cephalic bulb with **10-21** transverse rows of hooks…**2**
2. Cephalic bulb with more than **25** transverse rows of hooks…**3**
3. **11-13** transverse rows of hooks, **8** pairs of caudal papillae, vulva 0.6-0.65 mm from posterior end, from Indian Ocean (Ceylon), Myliobatiformes…*E. multidentatus*
4. **14-21** transverse rows of hooks…**4**
5. **25-30** transverse rows…**5**
6. **More than 30** transverse rows…**6**
7. **16-21** transverse rows, **6** pairs of caudal papillae, vulva **2.53-3.52** mm from posterior end, from South America (Mexico), Heterodontiformes…*E. pseudouncinatus*
8. **15-18** transverse rows, **8** pairs of caudal papillae, vulva **0.55** mm from posterior end, from Indian Ocean (Ceylon), Myliobatiformes…*E. southwelli*
9. **5-7** pairs of caudal papillae, Oe/BL ratio **15-20%**, from Southern China and New Caledonia, Myliobatiformes…*E. pteroplateae*
10. **8** pairs of caudal papillae, Oe/BL ratio **8-12%**, from Venezuela, Myliobatiformes…*E. diazi*
11. **7** pairs of caudal papillae (rarely 8**)**…**7**
12. **8-9** pairs of caudal papillae…**8**
13. **Cervical sac of unequal size, 7** (rarely 8) pairs of caudal papillae, Oe/BL ratio **11-16%**, spicules unequal, spicules/BL ratio **2.0-5.0%**, gubernaculum present, from Australia, French Polynesia and New Caledonia, Heterodontiformes, Myliobatiformes, Orectolobiformes, Rajiformes, Rhinopristiformes, Torpediniformes and Chimaeriformes…*E. overstreeti*
14. **Cervical sacs of equal size, 7** pairs of cervical papillae…**9**
15. **8** pairs of caudal papillae…**10**
16. **9** pairs of cervical papillae…**11**
17. Interlabia **with distinct cuticular striation, 7** pairs of caudal papillae, Oe/BL ratio **16-22%**, Spicules/BL ratio **3.0-3.1%**, Gubernaculum present, from Tunisia, Carcharhiniformes…*E. caniculus* **n. sp.**
18. Interlabia **without distinct cuticular striation, 7** pairs of caudal papillae, Oe/BL ratio **17-20**%, spicules almost equal, spicules/BL ratio **4.5-9.2%**, Gubernaculum present, from New Caledonia, Myliobatiformes…*E. inserratus*
19. **8** pairs of caudal papillae, spicules/BL ratio **10-14.3%** (in contrast to 7a), spicules **unequal**, area rugosa **present**, vulva **1.4-2.**5 mm from posterior end, from Adriatic and Black Sea, Myliobatiformes…*E. uncinatus*
20. **8** pairs of caudal papillae, spicules/BL ratio **6.6-7.4%** (in contrast to 7a), spicules **equal**, area rugosa **absent**, vulva **1.1-1.4** mm from posterior end, from Indian Ocean, Myliobatiformes…*E. spinosissimus*
21. **9** pairs of caudal papillae, body length **55-65mm**, spicules **equal**, from Central and South America, Myliobatiformes…*E. daileyi*
22. **9** pairs of caudal papillae, body length **13-33mm**, spicules **unequal**, reported from Central and South America, Myliobatiformes…*E. janzeni*

## Conclusion

By comparing the morphology of adult male and female *Echinocephalus* specimens from *Scyliorhinus canicula* collected off Tunisia with the descriptions of all congeners, it is evident that the collected specimens represent a new species, *E. caniculus* **n. sp.** The morphological description with notes on ecology, host specificity and zoogeography add new information to the knowledge of the parasite fauna of the lesser spotted dogfish in general, and in the Mediterranean basin sensu stricto, providing the first record of nematodes of the genus *Echinocephalus* in Tunisia.

## Data Availability

All data published within this text. Not applicable
